# Supplementary material for: A UMLS-based spell checker for natural language processing in vaccine safety
Source: BMC Med Inform Decis Mak. 2007 Feb 12;7:3. doi: 10.1186/1472-6947-7-3 (PMC1805499; doi:10.1186/1472-6947-7-3)
Supplement: Additional file 13 — RAP application directory. Sets up directory for RAP (RDF application for PHP) source code [file 1472-6947-7-3-S13.gz › rap/doc/phpdoc/model/Node.html]

xml version="1.0" encoding="iso-8859-1"?


Docs For Class Node


## Class Node

Description

Description |
Descendents
| Methods

An abstract RDF node.

Can either be resource, literal or blank node. Node is used in some comparisons like is\_a($obj, "Node"), meaning is $obj a resource, blank node or literal.

History:

- 09-10-2002 : First version of this class.

- abstract:
- todo: nothing
- author: Chris Bizer <chris@bizer.de>
- version: V0.9.1

Located in /model/Node.php (line 25)

```
Object
   |
   --Node
```

Direct descendents

Description |
Descendents
| Methods

| Class | Description |
| --- | --- |
| Literal | An RDF literal. |
| Resource | An RDF resource. |

Methods

Description |
Descendents |
Methods

#### Inherited Methods


Inherited From Object

> Object::toString()

Documentation generated on Thu, 7 Jul 2005 13:42:17 +0200 by phpDocumentor 1.3.0RC3
